# Trends in Avoidable Mortality in Kazakhstan From 2015 to 2021

**DOI:** 10.34172/ijhpm.2024.7919

**Published:** 2024-03-13

**Authors:** Lyazzat Kosherbayeva, Nazgul Akhtayeva, Kamshat Tolganbayeva, Aizhan Samambayeva

**Affiliations:** ^1^Asfendiyarov Kazakh National Medical University, Almaty, Kazakhstan.; ^2^Al-Farabi Kazakh National University, Almaty, Kazakhstan.; ^3^AyEconomics Research Center, Santiago, Spain.

**Keywords:** Mortality, Healthcare System, Trends, Health Outcomes, Health Services

## Abstract

**Background:** The health system performance assessment is a challenging process for decision-makers. In case of Kazakhstan’s healthcare system, the calculation of avoidable mortality, which has been underutilized to date, could serve as an additional tool to prioritize areas for improvement. Therefore, the aim of the study is to analyse avoidable mortality in Kazakhstan.

**Methods:** The data was retrieved from the Bureau of National Statistics, Kazakhstan. It covers population data by age, mortality rates from disease groups based on the Joint OECD (Organisation for Economic Co-operation and Development)/Eurostat classification of preventable and treatable causes of mortality. The data spans from 2015 to 2021, categorized by gender and 5-year age groups (0, 1-4, 5-9,..., 70-74). Standardization was performed using the 2015 OECD standard population. We used joinpoint regression analysis to calculate the average annual percentage change (AAPC).

**Results:** From 2015 to 2019, the annual percentage change (APC) in avoidable mortality per 100 000 population was -3.8 (-5.7 to -1.8), and from 2019 to 2021 it increased by 17.6 (11.3 to 24.3). Males exhibited higher avoidable mortality rates compared to females. The preventable mortality rate was consistently higher than the treatable mortality. Both preventable and treatable mortality decreased from 2015 to 2019, with preventable mortality reaching 272.17 before rising to 379.23 per 100 000 population in 2021. Between 2015 and 2021, treatable mortality rates increased from 179.3 (176.93-181.67) to 205.45 (203.08-207.81) per 100 000 population.

**Conclusion:** In Kazakhstan, the leading causes of avoidable mortality were circulatory diseases, respiratory diseases, and cancer. To achieve the goals of universal health coverage (UHC) and improve the overall population health, there is an urgent need to amend the healthcare system and reduce avoidable mortality. While it is important to acknowledge the influence of COVID-19 on these trends, our study’s focus on avoidable mortality provides valuable insights that complement the understanding of pandemic-related effects.

## Background

**Key Messages**

**Implications for policy makers**

- Enhance public health measures focusing on prevention, especially for circulatory and respiratory diseases, cancer, and injury prevention.
- Develop and implement health policies that address the higher avoidable mortality rates in males.
- Improve the healthcare infrastructure to ensure equitable access to quality medical care.
- Implement robust systems for continuous monitoring and analysis of health data to identify trends in preventable and treatable diseases.
- Enhance the healthcare system’s capacity to respond to health crises, like pandemics, which can significantly impact mortality rates.

**Implications for the public**

The findings of our research on avoidable mortality in Kazakhstan from 2015 to 2021 hold significant implications for the public. This study highlights the tangible impact that healthcare policies and personal health choices can have on population lives. For instance, higher rates of diseases related to circulatory and respiratory systems, cancer, and injuries especially in males, underscore the relevance of preventive healthcare practices. By understanding these trends, individuals can take proactive steps towards healthier lifestyles, such as regular health screening, balanced diet, and exercise, which are crucial in preventing these common but serious health issues. Additionally, the study’s results serve as a reminder of the critical role of robust healthcare services and the need for public advocacy for improved healthcare policies.

Health system performance assessment includes various internal processes for obtaining and using evidence and evaluating performance. Nowadays, there are various complementary tools for assessing the health system performance, such as the assessment of disability adjusted life year,^1^ of premature mortality^2^ as well as avoidable mortality.^3^

 Avoidable mortality is used as an indicator to quantify the effect of healthcare to improving population health^4^ and as a key indicator of broader health inequalities.^5^ The concept of “avoidable mortality” was introduced by Rutstein et al^6^ to assess the quality of medical care by identifying conditions in which death or disability should not occur if timely and effective medical care is provided. The list of diseases for which mortality could have been prevented was revised by Nolte and McKee,^7,8^ as well as by the Organization for Economic Co-operation and Development (OECD) and Eurostat expert group.^3^ Recently, Canada,^9^ Australia,^10^ and other OECD countries carried out calculations of avoidable mortality, enabling comparative analysis among countries as well as identifying weaknesses in healthcare systems and areas for deeper study.^11^ Furthermore, authors suggest using preventable and treatable mortality as indicative measures for public health and medical organizations^12^ in health system performance assessment documents. However, it is important to acknowledge that while these indicators provide insights into the impact of healthcare interventions on mortality rates, they may not cover the full spectrum of non-fatal health conditions and outcomes. In addition, it is essential to consider a broader range of health metrics to provide a comprehensive assessment of public health and healthcare systems. Moreover, existing approaches to measuring public health, such as smoking and alcohol consumption, may not always facilitate cross-country comparisons. In contrast, preventable mortality, however, can serve as a single, comprehensive indicator for this purpose.^12^

 Over the past two decades, Kazakhstan has undergone significant healthcare system reforms as part of its post-Soviet political and administrative transition.^13^ These reforms introduced clinical protocol development and regulation, health technology assessment (in particular, for implementation of new technologies), and a shift from the Beveridge model to a compulsory social health insurance system.^14-17^ Before the onset of the COVID-19 pandemic, positive trends were observed in several indicators. From 2013 to 2019, the death rate decreased from 8.0 to 7.1 per 1000 population, and life expectancy increased from 70.6 to 73.2 years. However, in 2021 the death rate rose to 9.6 per 1000 population, and life expectancy decreased to 70.2.^18^ Additionally, the population of Kazakhstan increased in 2013–2021 from 17.0 to 19.0 million.

 Kazakhstan supports the policy of universal health coverage (UHC) by strengthening the primary healthcare sector. To enhance healthcare systems further, effective measures have been implemented, including screening programs for early risk factor detection, disease management initiatives,^16^ health technology assessment processes,^15,17^ and promotion of the multidisciplinary team approach at primary healthcare. To enhance practical understanding of overcoming implementation barriers and strengthening primary healthcare, the World Health Organization Europe established the Primary Healthcare Demonstration Platform in Kazakhstan.^19^ Consequently, in assessing health system performance, the calculation of avoidable mortality, which has not been previously used, can serve as an additional tool for prioritizing areas of improvement. Therefore, the objective of this study was to examine trends in avoidable mortality in Kazakhstan from 2015 to 2021.

## Methods

###  Data Source

 The data was collected from the Bureau of National Statistics of the Agency for Strategic Planning and Reforms of the Republic of Kazakhstan (https://stat.gov.kz). Official statistical mortality data of all regions of Kazakhstan as of 10th revision of the International Statistical Classification of Diseases (ICD-10) between 2015 and 2021 was used to analyze avoidable mortality. The study population for analysis included a total of 633 109 deaths (364 420 for males and 268 689 for females) from 2015 to 2021.

 The comprehensive and precise mortality data is indispensable for conducting meaningful and representative assessments of health system performance. It’s worth noting that Kazakhstan launched the Unified Health Information System in 2012, which has significantly reduced the risk of inaccurate data.^20^ Available evidence indicates that the death registry in Kazakhstan is 90%–99% complete.^21^ Although concerns regarding the coverage and quality of death registries are acknowledged, a comprehensive quality assurance process was executed to guarantee the reliability of the data used in this manuscript. To validate the death registry data’s reliability, a comparative analysis was conducted by cross-referencing it with other pertinent sources of healthcare information, such as hospital records, vital statistics, and epidemiological studies, to identify and correct any potential discrepancies.

 In our research, in accordance with the OECD/Eurostat lists of preventable and treatable causes of death (January 2022 version), from ill-defined causes ICD-10 included Y16–Y34. For them we used the absolute numbers of deaths (presented by the Bureau of National Statistics, Kazakhstan), since we were not able to calculate ill-defined deaths in accordance with the World Health Organization (WHO) methodology^22^ due to lack of access to total deaths for the year under study.

###  Avoidable Mortality and Age Standardized Mortality Rate

 The concept of avoidable mortality draws its definition from well-respected sources, including the UK Office of National Statistics^23^ as well as OECD/Eurostat lists of preventable and treatable causes of death (January 2022 version).^3^ Based on the methodology, avoidable mortality serves as an indicator for assessing the extent to which premature deaths resulting from specific conditions ought to be infrequent and ideally prevented through timely and efficient healthcare interventions.^24^ Avoidable mortality consists of two components – the sum of preventable and treatable mortality.^3^ Preventable mortality pertains to factors leading to death that can be largely mitigated through successful implementation of public health strategies and primary preventive actions. These interventions are focused on reducing the occurrence of diseases or injuries. On the other hand, treatable mortality encompasses causes of death that can be significantly minimized through prompt and efficient healthcare interventions, encompassing both secondary prevention and effective treatment measures. This approach targets reducing the fatality rate after diseases have already manifested.^3^

 The selection of causes of death for the lists of preventable and treatable causes was taken from the OECD/Eurostat lists of preventable and treatable causes of death (January 2022 version). This list consists of 14 nosology groups and 95 causes of deaths.^3^ Age-standardized death rates are given in OECD 2015, where the standardization is based on the 2015 OECD standard population, so that it can be used for comparative analysis.^24^ The main causes of death were taken according to the ICD-10. Furthermore, data from 0–74-year-olds were included for the avoidable mortality calculation by gender and 5-year age group (0, 1-4, 5-9, 10-14,..., 70-74).

###  Statistical Analysis

 The absolute (per 100 000 population) and relative difference (%) in mortality were presented by gender and period. Corresponding 95% confidence intervals (95% CIs) were calculated by gender and age group. To identify changes in avoidable mortality ratio trends for the period 2015–2021, joinpoint regression was used by calculating the annual percentage change (APC) and average annual percentage change (AAPC, %) for every age and gender group by using the software Joinpoint 4.9.1.0 Regression Program.^25^ All statistical analyses were performed using Microsoft Excel and SPSS 13.

## Results

 Age-standardized avoidable mortality rates in Kazakhstan in 2015–2021 are shown in Table 1. Between 2015 and 2019, the age-adjusted avoidable mortality rate per 100 000 population fell from 505.37 (501.38; 509.36) to 432.55 (429.07; 436.03), and then rose to 587.95 (583.92; 591.98) in 2021. From 2015 to 2019, APC of avoidable mortality was -3.8 (-5.7; -1.8), increasing to 17.6 (11.3; 24.3) per 100 000 population in 2019-2021. The AAPC from 2015 to 2021 was 2.9 (1.8; 4.0) per 100 000 population. In all cause of mortality males had higher avoidable (preventable and treatable) mortality rates compared to females over the given period. However, females had higher avoidable mortality rates than males AAPC were 4.7 (2.9; 6.5) than males 1.8 (0.9; 2.2) per 100 000 population (Table 1).

**Table 1:** Age-Standardized Avoidable Mortality Rates (Per 100 000 Population; 95% Confidence Interval) by gender in Kazakhstan from 2015 to 2021

**Years**	**Avoidable**	**Preventable**	**Treatable**
**Total**			
2015	505.37 (501.38; 509.36)	326.07 (322.86; 329.28)	179.30 (176.93; 181.67)
2016	480.82 (476.96; 484.68)	308.74 (305.65; 311.84)	172.08 (169.77; 174.38)
2017	451.64 (447.94; 455.34)	287.51 (284.56; 290.46)	164.13 (161.9; 166.36)
2018	439.14 (435.56; 442.71)	277.68 (274.84; 280.53)	161.45 (159.29; 163.62)
2019	432.55 (429.07; 436.03)	272.17 (269.41; 274.94)	160.38 (158.27; 162.49)
2020	508.70 (504.94; 512.45)	292.83 (289.97; 295.68)	215.87 (213.43; 218.31)
2021	587.95 (583.92; 591.98)	382.50 (379.23; 385.77)	205.45 (203.08; 207.81)
APC	-3.8* (-5.7; -1.8) (*P* = .015)/2015-2019 17.6* (11.3; 24.3) (*P* = .006)/2019-2021	-5.3* (-7.3; -3.3) (*P* = .0008)/2015-2019 19.5* (12.8; 26.7) (*P* = .006)/2019-2021	-3.9 (-33.3; 38.4) (*P* = .682)/2015-2019 10.8 (-19.8; 53.0) (*P* = .306)/2019-2021
AAPC 2015-2021	2.9* (1.8; 4.0) (*P* < .001)	2.4* (1.2; 3.5) (*P* < .001)	3.2 (-7.7; 15.3) (*P* = .584)
**Male**			
2015	763.36 (755.93; 770.80)	525.66 (519.50; 531.82)	237.70 (233.54; 241.87)
2016	740.70 (733.40; 748.00)	508.43 (502.38; 514.47)	232.28 (228.18; 236.37)
2017	699.91 (692.86; 706.97)	473.50 (467.72; 479.28)	226.42 (222.37; 230.46)
2018	679.58 (672.76; 686.40)	457.38 (451.79; 462.96)	222.20 (218.29; 226.12)
2019	671.91 (665.25; 678.57)	449.71 (444.26; 455.16)	222.20 (218.37; 226.03)
2020	773.84 (766.71; 780.98)	476.29 (470.71; 481.87)	297.56 (293.11; 302.00)
2021	830.08 (822.77; 837.39)	563.47 (557.43; 569.50)	266.61 (262.49; 270.74)
APC	-3.0* (-4.6; -1.4) (*P* = .016)/2015-2019 12.1* (7.1; 17.3) (*P* = .009)/2019-2021	-4.5* (-6.7; -2.2) (*P* = .014)/2015-2019 12.7* (5.5; 20.4) (*P* = .016)/2019-2021	-2.2 (-36.0; 49.5) (*P* = .845)/2015-2019 8.6 (-25.4; 58.2) (*P* = .442)/2019-2021
AAPC 2015-2021	1.8* (0.9; 2.7) (*P* < .001)	0.9 (-0.3; 2.2) (*P* = .134)	3.1 (-9.4; 17.3) (*P* = .643)
**Female**			
2015	321.49 (317.15; 325.83)	183.25 (179.93; 186.56)	138.24 (135.45; 141.04)
2016	302.51 (298.33; 306.70)	170.26 (167.08; 173.45)	132.25 (129.53; 134.96)
2017	287.79 (283.75; 291.84)	162.30 (159.22; 165.38)	125.50 (122.87; 128.12)
2018	284.46 (280.49; 288.43)	159.05 (156.04; 162.06)	125.41 (122.82; 128.00)
2019	277.85 (274.02; 281.68)	154.73 (151.83; 157.63)	123.12 (120.61; 125.63)
2020	337.23 (333.07; 341.38)	170.82 (167.83; 173.81)	166.40 (163.52; 169.28)
2021	413.46 (408.94; 417.98)	251.87 (248.31; 255.44)	161.58 (158.81; 164.36)
APC	-3.5* (-6.8; 0.1) (*P* = .047)/2015-2019 23.1* (12.6; 34.6) (*P* = .01)/2019-2021	-5.3* (-8.7; -1.7) (*P* = .024)/2015-2019 29.5* (17.6; 42.6) (*P* = .007)/2019-2021	-4.0 ( -32.0; 35.6) (*P* = .664)/2015-2019 11.3 (-18.1; 51.3) (*P* = .271)/2019-2021
AAPC 2015-2021	4.7* (2.9; 6.5) (*P* < .001)	5.1* (3.2; 7.1) (*P* < .001)	3.4 (-6.9; 14.9) (*P* = .534)

Abbreviations: APC, annual percentage change; AAPC, average annual percentage change.

 The preventable mortality rate was higher compared to treatable mortality in this period. The APC of preventable mortality decreased from 2015 to 2019 by -5.3 (-7.3; -3.3) but later increased by 19.5 (12.8; 26.7). The AAPC between 2015 and 2021 has increased to 2.4 (1.2; 3.5) per 100 000 population. In terms of preventable mortality, the AAPC in females have increased five times more 5.1 (3.2; 7.1) compared to males 0.9 (-0.3; 2.2). In both groups, the preventable mortality decreased from 2015 to 2019 achieving 272.17 (269.41; 274.94), after which it rose to 382.5 (379.23; 385.77) in 2021 (Table 1).

 Between 2015 and 2021, treatable mortality rates increased from 179.3 (176.93; 181.67) to 205.45 (203.08; 207.81) per 100 000 population, with the lowest point in 2019 being 160.38 (158.27; 162.49). APC from 2015 to 2019 declined by -3.9 (-33.3; 38.4), while from 2019 to 2021 it increased to 10.8 (-19.8; 53.0). AAPC increased by 3.2 (-7.7; 15.3) per 100 000 population from 2015 to 2021. During 2015 and 2021, treatable mortality in males increased from 237.7 (233.54; 241.87) to 266.61 (262.49; 270.74), with the lowest point in 2018 being 222.2 (218.29; 226.12) and in 2019 it was 222.2 (218.37; 226.03) while the highest – in 2020, when APC was 297.56 (293.11; 302.00). The situation for females and males was similar, where the rate of treatable mortality decreased from 2015 to 2019, and then an increase was observed in 2021. However, APC between 2015 and 2019 decreased more in females -4.0 (-32.0; 35.6) than in males -2.2 (-36.0; 49.5), and afterwards females had a higher increase of 11.3 (-18.1; 51.3) compared to males 8.6 (-25.4; 58.2).

 From 2015 to 2021 the AAPC for males and females was similar 3.1 (-9.4; 17.3) and 3.4 (-6.9; 14.9), respectively (Table 1). The top five diseases with the highest preventable mortality were diseases of the circulatory system, cancer, respiratory system, injuries, and alcohol-related and drug-related deaths.

###  Changes of Avoidable Mortality by Groups, Causes of Deaths, and Gender

Table 2 presents the absolute and relative changes of cause-specific avoidable (preventable and treatable) mortality by gender between 2015 and 2021.

**Table 2:** Absolute and Relative Changes of Cause-Specific Avoidable Mortality Rate by Gender From 2015 to 2021

**Causes Group**	**Absolute Changes (Per 100 000 Population)**									**Relative Change (%)**								
	**Total**			**Male **			**Female **			**Total**			**Male **			**Female **		
	**Avoidable**	**Preventable**	**Treatable**	**Avoidable**	**Preventable**	**Treatable**	**Avoidable**	**Preventable**	**Treatable**	**Avoidable**	**Preventable**	**Treatable**	**Avoidable**	**Preventable**	**Treatable**	**Avoidable**	**Preventable**	**Treatable**
Infectious diseases	-1.39	-1.39	-0.01	-2.73	-2.24	-0.49	-0.53	-0.75	0.23	-15.32	-32.62	-0.11	-20.12	-33.95	-7.03	-9.51	-32.34	7.12
Tuberculosis	-2.35	-1.18	-1.18	-4.01	-2.01	-2.01	-1.08	-0.54	-0.54	-51.70	-51.70	-51.70	-51.48	-51.48	-51.48	-54.86	-54.86	-54.86
Others	-0.21	-0.21	0.00	-0.23	-0.23	0.00	-0.22	-0.22	0.00	-10.68	-10.68	0.00	-8.57	-8.57	0.00	-15.97	-15.97	0.00
Cancer	-22.03	-16.86	-5.17	-31.65	-28.61	-3.03	-16.81	-9.75	-7.06	-26.51	-30.16	-19.01	-28.21	-30.19	-17.42	-25.75	-32.50	-20.01
Stomach cancer	-3.98	-3.98	0.00	-5.92	-5.92	0.00	-2.85	-2.85	0.00	-27.70	-27.70	0.00	-25.31	-25.31	0.00	-34.11	-34.11	0.00
Liver cancer	-1.81	-1.81	0.00	-2.39	-2.39	0.00	-1.49	-1.49	0.00	-32.40	-32.40	0.00	-29.32	-29.32	0.00	-39.12	-39.12	0.00
Lung cancer	-6.59	-6.59	0.00	-14.54	-14.54	0.00	-1.55	-1.55	0.00	-31.58	-31.58	0.00	-33.85	-33.85	0.00	-25.94	-25.94	0.00
Colorectal cancer	-2.62	0.00	-2.62	-2.49	0.00	-2.49	-2.78	0.00	-2.78	-23.86	0.00	-23.86	-18.27	0.00	-18.27	-29.91	0.00	-29.91
Breast cancer	-1.67	0.00	-1.67	0.00	0.00	0.00	-2.98	0.00	-2.98	-18.39	0.00	-18.39	0.00	0.00	0.00	-18.52	0.00	-18.52
Cervical cancer	-0.77	-0.39	-0.39	0.00	0.00	0.00	-1.49	-0.74	-0.74	-18.33	-18.33	-18.33	0.00	0.00	0.00	-19.14	-19.14	-19.14
Others	-4.58	-4.09	-0.49	-6.30	-5.76	-0.54	-3.67	-3.12	-0.55	-25.49	-31.56	-9.79	-26.21	-28.41	-14.34	-26.26	-39.05	-9.21
Endocrine and metabolic diseases	13.50	7.00	6.50	14.01	7.15	6.86	13.21	6.94	6.27	61.51	65.69	57.57	62.94	65.18	60.75	60.99	66.83	55.62
Diabetes mellitus	13.92	6.96	6.96	14.23	7.11	7.11	13.76	6.88	6.88	66.33	66.33	66.33	65.82	65.82	65.82	67.22	67.22	67.22
Others	-0.42	0.04	-0.46	-0.21	0.04	-0.25	-0.56	0.06	-0.62	-44.53	23.93	-58.24	-33.15	23.20	-52.03	-47.26	39.99	-59.85
Diseases of the nervous system (epilepsy)	-0.08	0.00	-0.08	-0.24	0.00	-0.24	0.03	0.00	0.03	-4.32	0.00	-4.32	-9.79	0.00	-9.79	2.90	0.00	2.90
Diseases of the circulatory system	16.42	6.58	9.84	19.34	7.42	11.92	13.27	5.52	7.75	10.84	9.02	12.53	8.51	6.73	10.18	13.59	11.88	15.16
Ischemic heart diseases	12.23	6.12	6.12	9.70	4.85	4.85	13.73	6.86	6.86	17.68	17.68	17.68	8.44	8.44	8.44	37.73	37.73	37.73
Cerebrovascular diseases	3.29	1.64	1.64	8.88	4.44	4.44	-1.29	-0.65	-0.65	5.14	5.14	5.14	10.24	10.24	10.24	-2.69	-2.69	-2.69
Others	0.90	-1.18	2.08	0.77	-1.86	2.63	0.84	-0.69	1.53	4.91	-18.25	17.43	2.97	-19.62	16.13	6.33	-16.18	17.18
Diseases of the respiratory system	7.44	-4.00	11.44	2.84	-6.76	9.60	11.03	-1.91	12.94	9.11	-6.95	47.42	2.13	-7.06	25.31	24.09	-5.98	93.69
Chronic lower respiratory diseases	-3.90	-3.90	0.00	-6.55	-6.55	0.00	-1.87	-1.87	0.00	-6.83	-6.83	0.00	-6.91	-6.91	0.00	-5.89	-5.89	0.00
Pneumonia, not elsewhere classified	10.83	0.00	10.83	8.74	0.00	8.74	12.55	0.00	12.55	54.13	0.00	54.13	27.06	0.00	27.06	116.20	0.00	116.20
Others	0.50	-0.11	0.60	0.65	-0.20	0.85	0.35	-0.05	0.40	10.85	-21.76	14.70	10.25	-27.27	15.22	10.54	-15.10	13.19
Diseases of the digestive system	-0.41	0.00	-0.41	-0.57	0.00	-0.57	-0.31	0.00	-0.31	-3.48	0.00	-3.48	-3.22	0.00	-3.22	-4.25	0.00	-4.25
Gastric and duodenal ulcer	-0.29	0.00	-0.29	-0.60	0.00	-0.60	-0.05	0.00	-0.05	-6.56	0.00	-6.56	-8.23	0.00	-8.23	-2.00	0.00	-2.00
Others	-0.12	0.00	-0.12	0.03	0.00	0.03	-0.26	0.00	-0.26	-1.64	0.00	-1.64	0.28	0.00	0.28	-5.30	0.00	-5.30
Diseases of the genitourinary system	4.48	0.00	4.48	6.41	0.00	6.41	2.98	0.00	2.98	39.13	0.00	39.13	36.92	0.00	36.92	38.55	0.00	38.55
Renal failure	4.39	0.00	4.39	6.70	0.00	6.70	2.65	0.00	2.65	64.96	0.00	64.96	83.01	0.00	83.01	44.60	0.00	44.60
Others	0.10	0.00	0.10	-0.29	0.00	-0.29	0.34	0.00	0.34	2.03	0.00	2.03	-3.18	0.00	-3.18	18.67	0.00	18.67
Diseases of pregnancy, childbirth, and perinatal period	0.07	0.00	0.07	-0.94	0.00	-0.94	1.01	0.00	1.01	1.11	0.00	1.11	-13.25	0.00	-13.25	18.08	0.00	18.08
Certain conditions originating in the perinatal period	-0.30	0.00	-0.30	-0.94	0.00	-0.94	0.28	0.00	0.28	-4.84	0.00	-4.84	-13.25	0.00	-13.25	5.27	0.00	5.27
Others	0.37	0.00	0.37	0.00	0.00	0.00	0.73	0.00	0.73	217.14	0.00	217.14	0.00	0.00	0.00	217.00	0.00	217.00
Congenital malformations	-0.33	0.04	-0.37	-0.26	0.03	-0.29	-0.43	0.06	-0.49	-18.05	148.87	-20.61	-13.19	60.87	-14.82	-24.71	468.33	-28.36
Congenital malformations of the circulatory system	-0.37	0.00	-0.37	-0.29	0.00	-0.29	-0.49	0.00	-0.49	-20.61	0.00	-20.61	-14.82	0.00	-14.82	-28.36	0.00	-28.36
Others	0.04	0.04	0.00	0.03	0.03	0.00	0.06	0.06	0.00	148.87	148.87	0.00	60.87	60.87	0.00	468.33	468.33	0.00
Adverse effects of medical and surgical care	-0.08	0.00	-0.08	-0.16	0.00	-0.16	-0.01	0.00	-0.01	-44.08	0.00	-44.08	-65.62	0.00	-65.62	-11.61	0.00	-11.61
Misadventures to patients during surgical and medical care	-0.02	0.00	-0.02	-0.09	0.00	-0.09	0.02	0.00	0.02	-48.82	0.00	-48.82	-85.28	0.00	-85.28	170.40	0.00	170.40
Others	-0.05	0.00	-0.05	-0.08	0.00	-0.08	-0.03	0.00	-0.03	-42.21	0.00	-42.21	-52.34	0.00	-52.34	-31.74	0.00	-31.74
Injuries	-17.84	-17.84	0.00	-31.86	-31.86	0.00	-7.04	-7.04	0.00	-23.92	-23.92	0.00	-24.87	-24.87	0.00	-24.15	-24.15	0.00
Transport accidents	-2.48	-2.48	0.00	-4.13	-4.13	0.00	-1.23	-1.23	0.00	-16.51	-16.51	0.00	-17.38	-17.38	0.00	-16.62	-16.62	0.00
Intentional self-harm	-4.92	-4.92	0.00	-8.78	-8.78	0.00	-1.66	-1.66	0.00	-29.83	-29.83	0.00	-29.96	-29.96	0.00	-30.90	-30.90	0.00
Others	-10.43	-10.43	0.00	-18.95	-18.95	0.00	-4.14	-4.14	0.00	-24.24	-24.24	0.00	-25.25	-25.25	0.00	-25.34	-25.34	0.00
Alcohol-related and drug-related deaths	-0.82	-0.82	0.00	-2.16	-2.16	0.00	-0.18	-0.18	0.00	-1.71	-1.71	0.00	-2.94	-2.94	0.00	-0.64	-0.64	0.00
Alcohol specific disorders and poisonings	0.35	0.35	0.00	-0.28	-0.28	0.00	0.31	0.31	0.00	0.76	0.76	0.00	-0.39	-0.39	0.00	1.13	1.13	0.00
Drug-related deaths	-1.17	-1.17	0.00	-1.89	-1.89	0.00	-0.49	-0.49	0.00	-63.32	-63.32	0.00	-66.56	-66.56	0.00	-53.59	-53.59	0.00
Provisional assignment of new diseases – COVID-19	65.83	65.83	0.00	70.65	70.65	0.00	61.95	61.95	0.00	364.13	364.13	0.00	281.38	281.38	0.00	438.81	438.81	0.00

 For preventable mortality, the greatest *absolute changes reduction *per 100 000 population was for cancer (-16.86), followed by injuries (-17.84); while the greatest increase was for endocrine and metabolic diseases (7.0) and diseases of the circulatory system (6.58). Drug-related deaths (-63.32), tuberculosis (-51.70), intentional self-harm (-29.83), and cancer (-30.16) had the greatest *relative changes reduction* in preventable mortality, while congenital malformations (148.87), endocrine and metabolic diseases (65.69) had the greatest relative increase.

 For majority of diseases, males had higher *absolute changes reduction in avoidable mortality* than females. For preventable mortality, ischemic heart disease has increased more in females (6.86) compared to males (4.85); by contrast, cerebrovascular diseases have increased more in males (4.44) compared to females (-0.65). Moreover, *absolute changes decrease* in preventable cancer (-28.61 and -9.75) and injuries (-31.86 and -7.04) was observed in males compared to females. The areas with the greatest differences in *relative changes* between genders were congenital malformations, drug-related deaths, liver cancer, ischemic heart disease, and cerebrovascular diseases.

 Regarding the treatable mortality, the highest *absolute changes decrease* was in cancer (-5.17 per 100 000 population) compared to other causes; by contrast, the greatest increase was in respiratory system diseases (11.44) and circulatory system diseases (9.84). In terms of treatable mortality, tuberculosis (-51.70), patient misadventures during surgical and medical care (-48.82), and adverse effects of medical and surgical care (-44.08) had the greatest *relative changes reductions*, while renal failure (64.96), endocrine and metabolic diseases (57.57) had the greatest increases. The *relative changes increased* more in females than males for respiratory system diseases (93.69 and 25.31, respectively).

 A new category of disease group was added by the OECD to the international definition of avoidable mortality to include COVID-19 as a preventable cause of death since most of these infections and deaths could be prevented through preventive measures such as vaccination.^3^ When comparing 2021 to 2020, COVID-19 preventable mortality increased fourfold, from 18.08 to 83.91 per 100 000 population, with males having higher rate than females (Supplementary file 1). Table 2 presents the absolute and relative changes in cause-specific avoidable mortality rates from 2015 to 2021 by gender. The results on preventable and treatable mortality by gender are shown in Table S1.

## Discussion

 This is the first analysis of avoidable mortality (preventable and treatable) for Kazakhstan using the OECD avoidable mortality methodology. Despite the implementation of UHC policies and the strengthening of primary healthcare, high rates of preventable and treatable mortality persist in Kazakhstan compared to European countries.^24^ In line with other countries, our findings revealed that avoidable mortality was higher in men than in women.^26^

 In Kazakhstan, the difference in life expectancy between males (66.3) and females (74.03) was 8 years in 2021.^18^ Studies revealed that in Kazakhstan, males utilized healthcare services less frequently,^27^ whereas females exhibited higher adherence to treatment and achieved better health outcomes than males.^28^ Addressing the underlying factors contributing to the increased mortality rates among males is a crucial concern in the country.

 For most diseases, the avoidable mortality rate in Kazakhstan decreased between 2015 and 2019, especially for diseases of circulatory and respiratory systems, cancer, and injuries. This phenomenon may be due to the implementation of comprehensive screening programs in outpatient settings in Kazakhstan.^29^ These programs are designed to facilitate the early detection of a range of health conditions, including arterial hypertension, coronary heart disease, diabetes mellitus, glaucoma, and behavioral risk factors. Additionally, they aim to identify viral hepatitis B and C infections and focus on early detection of various oncological diseases, such as cervical cancer, breast cancer, and colorectal cancer. This proactive approach to healthcare and early intervention may contribute to the observed differences in healthcare utilization and health outcomes between males and females in the country.^29,30^ Other studies have reported a decline in the mortality rate for oncological and cardiovascular diseases in Kazakhstan.^31-34^ Furthermore, over the last decade, Kazakhstan has witnessed the implementation of innovative technologies, which may have contributed to the reduction in mortality rates.^35^ Another potential reason for the decline in avoidable mortality could be linked to the introduction of disease management programs, specifically for hypertension, type 2 diabetes, and chronic heart failure.^15^ A World Bank report details positive results from the implementation of DMPs in Kazakhstan from 2013 to 2017, where 75% of patients with hypertension had their blood pressure stabilized, patients with diabetes performed better, and patients with heart failure had fewer hospitalizations.^15^

 Other European countries,^24,36^ as well as in Korea,^37^ in the United States,^38^ Iran,^39^ and Sweden^26^ have seen a similar decrease in avoidable mortality rates. However, in Kazakhstan, avoidable mortality has increased in 2020 and 2021, particularly due to diseases of the circulatory and respiratory systems, and preventable mortality from COVID-19. It was found that excess mortality due to the COVID-19 globally 120.3 deaths per 100 000 population.^40^ Moreover, it must be noted that COVID-19 had influence on reduction of the life expectancy, for instance, in Madrid – by 1.9 years for males and 1.6 years for females,^41^ in Iran – by 1.4 years in 2020,^42^ in Brazil – by 1.94 years in 2020.^43^

 The avoidable mortality due to alcohol- and drug-related disorders, including poisonings, has not changed in Kazakhstan. Choi et al^37^ found that avoidable mortality from endocrine and metabolic diseases has reduced in Korea, while in Kazakhstan has increased in both genders between 2015 and 2021. This fact occurs despite the introduction of screening programs for obesity. Managers need to study the causes and conduct a deeper analysis. Kulkaeva and colleagues^44^ previously determined low medical literacy among the population; possibly, this fact is true and primary care should raise more concerns about this issue.

 In 2020, the preventable mortality rate per 10 000 population due to COVID-19 was 36.1 in Wales, 28.5 in Scotland, 34.9 in a England.^45^ In Kazakhstan, however, this rate has risen to 83.9 in 2021. A different study identified a high mortality rate due to COVID-19 among males; this result is consistent with ours, where preventive mortality among men was higher compared to females.^41,46,47^

 Further studies are needed, including stratified data by rural and urban residence and life expectancy indicators, which will provide additional valuable information for decision-makers. A study of the relationship between avoidable mortality and the social characteristics of population^37,48,49^ would provide a deeper understanding of the current situation of health of the population, as well as revision of public health measures implemented in preventive and treatable conditions.

 It is important to acknowledge several limitations associated of this study. The study’s timeframe (2015–2021) may not capture the longer-term trends in avoidable mortality. Potentially limiting the ability to draw conclusive insights about the effectiveness of health strategies beyond this period. By the classification of the WHO, data are considered medium quality for Kazakhstan. Given the definition of usability and the stated rates, one can describe the accuracy of death registration data in Kazakhstan as “relatively accurate and of good quality.” High completeness rate, combined with a substantial proportion of meaningful causes, suggests that the data is capturing and reporting deaths with medically certified and meaningful cause-of-death information. We acknowledge that in many developing countries, including Kazakhstan, the coverage and quality of death registry data can be suboptimal. Factors such as underreporting of deaths, incomplete data capture, and inaccuracies in death records can affect the reliability and generalizability of our findings. While we have employed statistical methods to mitigate the impact of these limitations, it is important for readers to bear in mind that such adjustments may not fully compensate for the underlying data quality issues. Consequently, our results should be interpreted with caution, particularly when extrapolating towards contexts with different health information systems or when comparing across countries with varying degrees of registry completeness and accuracy.

 Based on the WHO methodology, ill-defined causes cover ICD-10 codes: R00-R94, R96-R99, Y10-Y34, Y87.2, C76, C80, C97, I47.2, I49.0, I46, I50, I51. 4, I51.5, I51.6, I51.9, I70.9, and is determined by the percentage of total deaths that has been assigned to ill-defined causes. However, the fact that we were able to obtain absolute data without applying this methodology indicates the need for further improvement of the system.

 While death registration data is generally considered the most reliable source for understanding the causes of death, it exhibits significant limitations, even within well-operating systems featuring medical certification of the cause of death. These limitations encompass the presence of “garbage codes” in certain countries, which represent a notable share of recorded deaths. Reassigning these deaths to accurate causes remains highly uncertain and often lacks empirical grounding. The determination of the underlying cause of death is influenced by the information available on the death certificate and can be notably affected by the sequence in which diagnoses are recorded. Variability in cause assignment due to differences in physician practices when certifying deaths remains largely unaddressed for most causes of death. Additionally, certain diseases and injuries present challenges in establishing causal relationships for the underlying cause, as seen in cases like diabetes and heart disease or Alzheimer’s disease, as well as drug or alcohol overdoses. Inaccuracies or underreporting of avoidable mortality cases could potentially influence the study’s outcomes.

## Conclusion

 In Kazakhstan, the primary contributors to avoidable mortality between 2015 and 2021 were diseases related to circulatory and respiratory systems, cancer, and injuries, with notably higher rates among males compared to females. The avoidable mortality rate in Kazakhstan exceeded that of most European countries. The application of avoidable mortality, a previously underutilized metric, has demonstrated its potential as an additional instrument for evaluating areas in need of improvement within the health system of Kazakhstan. Through the analysis of avoidable mortality data from 2015 to 2021, this research contributes to a more comprehensive understanding of key areas where targeted interventions can be implemented to drive positive changes in the country’s healthcare strategies. Improving the healthcare system is required to fully implement UHC policies and enhance the health of the population by reducing avoidable mortality. While acknowledging the impact of COVID-19 on these trends is crucial, our study’s emphasis on avoidable mortality offers valuable insights that complement the understanding of pandemic-related effects.

## Ethical issues

 Ethical approval for this type of study is not required by our institute.

## Competing interests

 Authors declare that they have no competing interests.

## Funding

 This research has been funded by the Science Committee of the Ministry of Education and Science of the Republic of Kazakhstan (Grant No. AP09058136 “Technologies for Performance Assessment and Impact Analysis of the Health Systems. Comparative analysis at International Level”).

## Supplementary files

Supplementary file 1 contains Table S1.
