# Supplementary material for: Trends in Avoidable Mortality in Kazakhstan From 2015 to 2021
Source: Int J Health Policy Manag. 2024 Mar 13;13:7919. doi: 10.34172/ijhpm.2024.7919 (PMC11608278; doi:10.34172/ijhpm.2024.7919)
Supplement: Supplementary file 1 — contains Table S1. [file ijhpm-13-7919-s001.pdf]

Article title: Trends in Avoidable Mortality in Kazakhstan From 2015 to 2021

Journal name: International Journal of Health Policy and Management (IJHPM)

Authors' information: Lyazzat Kosherbayeva[1,2]*, Nazgul Akhtayeva[1], Kamshat Tolganbayeva[1], Aizhan Samambayeva[3]

***Correspondence to:** Lyazzat Kosherbayeva; Email: kosherbaeva.l@kaznmu.kz

[1]Asfendiyarov Kazakh National Medical University, Almaty, Kazakhstan.

[2]Al-Farabi Kazakh National University, Almaty, Kazakhstan.

[3]AyEconomics Research Center, Santiago, Spain.

Citation: Kosherbayeva L, Akhtayeva N, Tolganbayeva K, Samambayeva A. Trends in avoidable mortality in Kazakhstan from 2015 to 2021. Int J Health Policy Manag. 2024;13:7919. doi:10.34172/ijhpm.2024.7919

Supplementary file 1

Table S1. Age standardized avoidable mortality rates (per 100.000 persons. 95% confidence interval) by gender and diseases in Kazakhstan from 2015 to 2021

Causes group	Years	Total			Male			Female		
		Avoidable	Preventable	Treatable	Avoidable	Preventable	Treatable	Avoidable	Preventable	Treatable
**Infectious diseases**	**2015**	9.10	4.26	4.84	13.56	6.59	6.97	5.53	2.33	3.20
	**2016**	8.09	3.84	4.25	11.76	5.89	5.87	5.02	2.10	2.93
	**2017**	8.14	3.81	4.33	11.74	5.88	5.86	5.22	2.06	3.16
	**2018**	7.37	3.54	3.83	10.88	5.45	5.43	4.52	1.96	2.55
	**2019**	7.57	3.42	4.15	10.95	5.06	5.88	4.91	2.06	2.85
	**2020**	6.86	2.89	3.97	9.96	4.49	5.47	4.40	1.57	2.83
	**2021**	7.71	2.87	4.84	10.83	4.35	6.48	5.00	1.58	3.43
Tuberculosis	**2015**	4.55	2.28	2.28	7.80	3.90	3.90	1.96	0.98	0.98
	**2016**	3.66	1.83	1.83	5.98	2.99	2.99	1.76	0.88	0.88
	**2017**	3.45	1.73	1.73	5.71	2.86	2.86	1.65	0.83	0.83
	**2018**	2.88	1.44	1.44	4.86	2.43	2.43	1.28	0.64	0.64
	**2019**	2.56	1.28	1.28	4.27	2.14	2.14	1.23	0.62	0.62
	**2020**	2.27	1.14	1.14	3.80	1.90	1.90	1.08	0.54	0.54
	**2021**	2.20	1.10	1.10	3.78	1.89	1.89	0.89	0.44	0.44
Others	**2015**	4.54	1.98	2.56	5.76	2.69	3.07	3.57	1.35	2.22
	**2016**	4.43	2.01	2.42	5.78	2.90	2.88	3.27	1.22	2.05
	**2017**	4.69	2.08	2.61	6.03	3.03	3.00	3.57	1.24	2.33
	**2018**	4.48	2.10	2.39	6.03	3.02	3.00	3.23	1.32	1.91
	**2019**	5.00	2.14	2.86	6.67	2.92	3.75	3.68	1.44	2.24

	**2020**	4.59	1.76	2.83	6.16	2.59	3.57	3.32	1.03	2.29
	**2021**	5.51	1.77	3.74	7.05	2.46	4.59	4.12	1.13	2.98
**Cancer**	**2015**	83.09	55.89	27.20	112.18	94.77	17.40	65.29	30.01	35.28
	**2016**	77.84	51.80	26.04	106.99	89.88	17.10	60.52	26.78	33.74
	**2017**	72.12	47.14	24.98	97.24	80.10	17.14	58.17	25.99	32.18
	**2018**	69.47	45.74	23.72	95.87	80.26	15.61	55.43	24.09	31.34
	**2019**	66.32	43.59	22.73	92.16	76.52	15.64	52.32	22.86	29.46
	**2020**	64.81	42.53	22.28	88.54	73.37	15.17	51.67	22.77	28.90
	**2021**	61.07	39.04	22.03	80.53	66.16	14.37	48.48	20.26	28.22
Stomach cancer	**2015**	14.38	14.38	0.00	23.38	23.38	0.00	8.36	8.36	0.00
	**2016**	13.03	13.03	0.00	22.10	22.10	0.00	7.01	7.01	0.00
	**2017**	11.69	11.69	0.00	18.96	18.96	0.00	6.98	6.98	0.00
	**2018**	11.71	11.71	0.00	19.75	19.75	0.00	6.72	6.72	0.00
	**2019**	11.28	11.28	0.00	18.94	18.94	0.00	6.47	6.47	0.00
	**2020**	10.70	10.70	0.00	18.12	18.12	0.00	5.84	5.84	0.00
	**2021**	10.40	10.40	0.00	17.46	17.46	0.00	5.50	5.50	0.00
Liver cancer	**2015**	5.58	5.58	0.00	8.15	8.15	0.00	3.81	3.81	0.00
	**2016**	5.20	5.20	0.00	7.68	7.68	0.00	3.55	3.55	0.00
	**2017**	4.53	4.53	0.00	6.43	6.43	0.00	3.36	3.36	0.00
	**2018**	4.43	4.43	0.00	6.64	6.64	0.00	3.10	3.10	0.00
	**2019**	4.48	4.48	0.00	7.28	7.28	0.00	2.71	2.71	0.00
	**2020**	4.23	4.23	0.00	6.81	6.81	0.00	2.55	2.55	0.00
	**2021**	3.77	3.77	0.00	5.76	5.76	0.00	2.32	2.32	0.00
Lung cancer	**2015**	20.86	20.86	0.00	42.96	42.96	0.00	5.97	5.97	0.00
	**2016**	19.52	19.52	0.00	39.74	39.74	0.00	5.97	5.97	0.00
	**2017**	18.13	18.13	0.00	37.13	37.13	0.00	5.48	5.48	0.00
	**2018**	17.38	17.38	0.00	36.26	36.26	0.00	5.02	5.02	0.00
	**2019**	16.23	16.23	0.00	33.63	33.63	0.00	4.88	4.88	0.00
	**2020**	15.98	15.98	0.00	32.25	32.25	0.00	5.30	5.30	0.00
	**2021**	14.28	14.28	0.00	28.42	28.42	0.00	4.42	4.42	0.00
Colorectal cancer	**2015**	10.99	0.00	10.99	13.64	0.00	13.64	9.30	0.00	9.30
	**2016**	10.25	0.00	10.25	12.96	0.00	12.96	8.67	0.00	8.67
	**2017**	9.84	0.00	9.84	13.14	0.00	13.14	8.02	0.00	8.02
	**2018**	8.82	0.00	8.82	11.65	0.00	11.65	7.30	0.00	7.30
	**2019**	9.17	0.00	9.17	12.35	0.00	12.35	7.48	0.00	7.48
	**2020**	9.42	0.00	9.42	12.06	0.00	12.06	8.06	0.00	8.06
	**2021**	8.36	0.00	8.36	11.15	0.00	11.15	6.52	0.00	6.52

Breast cancer	**2015**	9.10	0.00	9.10	0.00	0.00	0.00	16.10	0.00	16.10
	**2016**	8.55	0.00	8.55	0.00	0.00	0.00	15.29	0.00	15.29
	**2017**	8.23	0.00	8.23	0.00	0.00	0.00	14.77	0.00	14.77
	**2018**	8.23	0.00	8.23	0.00	0.00	0.00	14.91	0.00	14.91
	**2019**	7.22	0.00	7.22	0.00	0.00	0.00	13.06	0.00	13.06
	**2020**	6.76	0.00	6.76	0.00	0.00	0.00	12.24	0.00	12.24
	**2021**	7.42	0.00	7.42	0.00	0.00	0.00	13.12	0.00	13.12
Cervical cancer	**2015**	4.23	2.11	2.11	0.00	0.00	0.00	7.77	3.88	3.88
	**2016**	4.08	2.04	2.04	0.00	0.00	0.00	7.47	3.74	3.74
	**2017**	4.11	2.05	2.05	0.00	0.00	0.00	7.54	3.77	3.77
	**2018**	3.93	1.97	1.97	0.00	0.00	0.00	7.28	3.64	3.64
	**2019**	3.64	1.82	1.82	0.00	0.00	0.00	6.75	3.38	3.38
	**2020**	3.76	1.88	1.88	0.00	0.00	0.00	6.96	3.48	3.48
	**2021**	3.45	1.73	1.73	0.00	0.00	0.00	6.28	3.14	3.14
Others	**2015**	17.96	12.96	5.01	24.05	20.29	3.76	13.99	7.99	6.00
	**2016**	17.20	12.01	5.19	24.51	20.36	4.15	12.55	6.50	6.05
	**2017**	15.60	10.74	4.86	21.58	17.58	4.00	12.01	6.39	5.62
	**2018**	14.96	10.26	4.70	21.57	17.61	3.96	11.09	5.61	5.48
	**2019**	14.31	9.79	4.52	19.95	16.66	3.29	10.97	5.43	5.54
	**2020**	13.96	9.73	4.22	19.30	16.19	3.12	10.73	5.60	5.12
	**2021**	13.38	8.87	4.52	17.75	14.52	3.22	10.31	4.87	5.44
**Endocrine and metabolic diseases**	**2015**	21.94	10.65	11.29	22.27	10.97	11.29	21.65	10.39	11.27
	**2016**	23.63	11.61	12.02	23.63	11.77	11.87	24.06	11.70	12.36
	**2017**	23.26	11.36	11.90	23.40	11.56	11.84	24.01	11.63	12.39
	**2018**	25.46	12.52	12.94	26.99	13.35	13.64	26.02	12.73	13.29
	**2019**	26.01	12.79	13.23	27.76	13.73	14.03	26.41	12.94	13.48
	**2020**	32.51	16.12	16.39	35.37	17.63	17.75	32.09	15.84	16.25
	**2021**	35.44	17.65	17.79	36.28	18.12	18.16	34.86	17.33	17.53
Diabetes mellitus	**2015**	20.99	10.49	10.49	21.62	10.81	10.81	20.48	10.24	10.24
	**2016**	22.78	11.39	11.39	22.99	11.49	11.49	23.01	11.51	11.51
	**2017**	22.37	11.19	11.19	22.64	11.32	11.32	22.97	11.49	11.49
	**2018**	24.56	12.28	12.28	26.24	13.12	13.12	24.99	12.49	12.49
	**2019**	25.27	12.64	12.64	27.16	13.58	13.58	25.54	12.77	12.77
	**2020**	31.91	15.96	15.96	34.99	17.49	17.49	31.30	15.65	15.65
	**2021**	34.91	17.45	17.45	35.85	17.92	17.92	34.24	17.12	17.12
Others	**2015**	0.95	0.16	0.79	0.65	0.16	0.49	1.18	0.15	1.03
	**2016**	0.85	0.22	0.63	0.64	0.27	0.37	1.05	0.19	0.85

	**2017**	0.89	0.18	0.72	0.76	0.24	0.52	1.04	0.14	0.90
	**2018**	0.90	0.24	0.66	0.76	0.23	0.52	1.03	0.24	0.79
	**2019**	0.74	0.15	0.59	0.60	0.14	0.45	0.87	0.17	0.71
	**2020**	0.60	0.16	0.44	0.38	0.13	0.25	0.80	0.19	0.60
	**2021**	0.53	0.20	0.33	0.43	0.20	0.23	0.62	0.21	0.41
**Diseases of the nervous system (epilepsy)**	**2015**	1.74	0.00	1.74	2.41	0.00	2.41	1.20	0.00	1.20
	**2016**	2.00	0.00	2.00	2.76	0.00	2.76	1.41	0.00	1.41
	**2017**	1.57	0.00	1.57	2.16	0.00	2.16	1.10	0.00	1.10
	**2018**	1.54	0.00	1.54	2.22	0.00	2.22	1.04	0.00	1.04
	**2019**	1.71	0.00	1.71	2.50	0.00	2.50	1.12	0.00	1.12
	**2020**	1.61	0.00	1.61	2.01	0.00	2.01	1.30	0.00	1.30
	**2021**	1.66	0.00	1.66	2.17	0.00	2.17	1.23	0.00	1.23
**Diseases of the circulatory system**	**2015**	151.48	73.01	78.47	227.40	110.30	117.11	97.61	46.50	51.11
	**2016**	143.46	68.83	74.63	224.33	108.45	115.88	88.41	41.88	46.53
	**2017**	137.21	66.03	71.18	217.80	105.52	112.28	84.67	40.27	44.40
	**2018**	131.51	63.26	68.25	209.72	101.67	108.05	82.10	38.96	43.14
	**2019**	125.44	60.06	65.38	201.42	97.28	104.13	77.02	36.26	40.76
	**2020**	145.15	68.30	76.85	231.00	109.61	121.39	89.72	41.60	48.13
	**2021**	167.90	79.59	88.31	246.75	117.72	129.03	110.88	52.02	58.86
Ischemic heart diseases	**2015**	69.20	34.60	34.60	114.88	57.44	57.44	36.38	18.19	18.19
	**2016**	64.89	32.45	32.45	111.33	55.66	55.66	32.76	16.38	16.38
	**2017**	61.71	30.85	30.85	106.94	53.47	53.47	31.43	15.71	15.71
	**2018**	61.11	30.55	30.55	106.04	53.02	53.02	31.71	15.86	15.86
	**2019**	56.95	28.48	28.48	99.44	49.72	49.72	28.73	14.37	14.37
	**2020**	64.90	32.45	32.45	110.77	55.38	55.38	34.39	17.19	17.19
	**2021**	81.43	40.72	40.72	124.57	62.29	62.29	50.11	25.05	25.05
Cerebrovascular diseases	**2015**	63.93	31.96	31.96	86.71	43.36	43.36	48.03	24.01	24.01
	**2016**	60.59	30.29	30.29	86.77	43.38	43.38	43.23	21.62	21.62
	**2017**	60.52	30.26	30.26	88.84	44.42	44.42	42.70	21.35	21.35
	**2018**	56.72	28.36	28.36	83.25	41.63	41.63	40.85	20.43	20.43
	**2019**	54.95	27.47	27.47	81.58	40.79	40.79	38.88	19.44	19.44
	**2020**	62.51	31.25	31.25	93.33	46.66	46.66	43.34	21.67	21.67
	**2021**	67.21	33.61	33.61	95.59	47.80	47.80	46.73	23.37	23.37
Others	**2015**	18.35	6.44	11.91	25.82	9.50	16.32	13.20	4.30	8.91
	**2016**	17.98	6.09	11.88	26.24	9.40	16.84	12.42	3.89	8.54
	**2017**	14.99	4.92	10.07	22.02	7.63	14.39	10.54	3.21	7.33
	**2018**	13.68	4.34	9.34	20.43	7.03	13.40	9.53	2.67	6.86

	**2019**	13.54	4.11	9.42	20.40	6.77	13.63	9.41	2.46	6.95
	**2020**	17.74	4.60	13.15	26.90	7.56	19.34	12.00	2.74	9.26
	**2021**	19.25	5.27	13.99	26.59	7.64	18.95	14.04	3.60	10.44
**Diseases of the respiratory system**	**2015**	81.66	57.55	24.12	133.57	95.65	37.92	45.80	31.98	13.82
	**2016**	79.98	57.95	22.03	135.39	100.82	34.57	43.74	30.59	13.14
	**2017**	72.52	51.77	20.75	126.95	92.81	34.14	38.49	26.84	11.65
	**2018**	68.76	47.48	21.28	116.90	83.09	33.81	39.42	26.65	12.78
	**2019**	67.87	45.32	22.56	117.03	80.99	36.04	37.80	24.44	13.36
	**2020**	107.37	45.32	62.04	168.61	80.25	88.36	70.16	24.39	45.77
	**2021**	89.10	53.55	35.56	136.41	88.89	47.52	56.83	30.07	26.76
Chronic lower respiratory diseases	**2015**	57.06	57.06	0.00	94.91	94.91	0.00	31.67	31.67	0.00
	**2016**	57.58	57.58	0.00	100.28	100.28	0.00	30.32	30.32	0.00
	**2017**	51.49	51.49	0.00	92.34	92.34	0.00	26.70	26.70	0.00
	**2018**	47.17	47.17	0.00	82.55	82.55	0.00	26.50	26.50	0.00
	**2019**	45.04	45.04	0.00	80.65	80.65	0.00	24.19	24.19	0.00
	**2020**	45.08	45.08	0.00	79.88	79.88	0.00	24.25	24.25	0.00
	**2021**	53.17	53.17	0.00	88.35	88.35	0.00	29.80	29.80	0.00
Pneumonia. not elsewhere classified	**2015**	20.01	0.00	20.01	32.31	0.00	32.31	10.80	0.00	10.80
	**2016**	18.59	0.00	18.59	29.69	0.00	29.69	10.66	0.00	10.66
	**2017**	17.25	0.00	17.25	29.05	0.00	29.05	9.13	0.00	9.13
	**2018**	17.72	0.00	17.72	28.45	0.00	28.45	10.32	0.00	10.32
	**2019**	19.58	0.00	19.58	31.84	0.00	31.84	11.12	0.00	11.12
	**2020**	58.61	0.00	58.61	83.38	0.00	83.38	43.30	0.00	43.30
	**2021**	30.85	0.00	30.85	41.05	0.00	41.05	23.34	0.00	23.34
Others	**2015**	4.59	0.49	4.11	6.36	0.74	5.61	3.33	0.31	3.02
	**2016**	3.82	0.37	3.44	5.43	0.54	4.89	2.76	0.27	2.49
	**2017**	3.78	0.27	3.50	5.56	0.47	5.09	2.66	0.15	2.51
	**2018**	3.87	0.31	3.56	5.90	0.54	5.36	2.60	0.15	2.45
	**2019**	3.26	0.28	2.98	4.54	0.34	4.20	2.49	0.25	2.24
	**2020**	3.68	0.25	3.43	5.35	0.37	4.98	2.62	0.15	2.47
	**2021**	5.09	0.38	4.71	7.01	0.54	6.47	3.68	0.27	3.42
**Diseases of the digestive system**	**2015**	11.78	0.00	11.78	17.74	0.00	17.74	7.22	0.00	7.22
	**2016**	11.47	0.00	11.47	17.26	0.00	17.26	7.17	0.00	7.17
	**2017**	11.10	0.00	11.10	16.31	0.00	16.31	7.50	0.00	7.50
	**2018**	10.58	0.00	10.58	16.19	0.00	16.19	6.80	0.00	6.80
	**2019**	11.69	0.00	11.69	17.61	0.00	17.61	7.63	0.00	7.63
	**2020**	11.42	0.00	11.42	17.53	0.00	17.53	7.12	0.00	7.12

	**2021**	11.37	0.00	11.37	17.17	0.00	17.17	6.91	0.00	6.91
Gastric and duodenal ulcer	**2015**	4.40	0.00	4.40	7.28	0.00	7.28	2.29	0.00	2.29
	**2016**	3.70	0.00	3.70	6.21	0.00	6.21	1.93	0.00	1.93
	**2017**	3.97	0.00	3.97	6.57	0.00	6.57	2.24	0.00	2.24
	**2018**	4.01	0.00	4.01	6.79	0.00	6.79	2.22	0.00	2.22
	**2019**	4.06	0.00	4.06	6.82	0.00	6.82	2.24	0.00	2.24
	**2020**	4.16	0.00	4.16	7.24	0.00	7.24	2.08	0.00	2.08
	**2021**	4.11	0.00	4.11	6.68	0.00	6.68	2.25	0.00	2.25
Others	**2015**	7.38	0.00	7.38	10.46	0.00	10.46	4.92	0.00	4.92
	**2016**	7.77	0.00	7.77	11.05	0.00	11.05	5.24	0.00	5.24
	**2017**	7.13	0.00	7.13	9.74	0.00	9.74	5.26	0.00	5.26
	**2018**	6.57	0.00	6.57	9.41	0.00	9.41	4.58	0.00	4.58
	**2019**	7.63	0.00	7.63	10.80	0.00	10.80	5.39	0.00	5.39
	**2020**	7.27	0.00	7.27	10.30	0.00	10.30	5.04	0.00	5.04
	**2021**	7.26	0.00	7.26	10.49	0.00	10.49	4.66	0.00	4.66
**Diseases of the genitourinary system**	**2015**	11.46	0.00	11.46	17.36	0.00	17.36	7.74	0.00	7.74
	**2016**	11.91	0.00	11.91	18.39	0.00	18.39	7.92	0.00	7.92
	**2017**	11.56	0.00	11.56	19.36	0.00	19.36	6.90	0.00	6.90
	**2018**	12.41	0.00	12.41	19.43	0.00	19.43	8.48	0.00	8.48
	**2019**	12.13	0.00	12.13	18.86	0.00	18.86	8.33	0.00	8.33
	**2020**	14.19	0.00	14.19	22.05	0.00	22.05	9.67	0.00	9.67
	**2021**	15.94	0.00	15.94	23.77	0.00	23.77	10.72	0.00	10.72
Renal failure	**2015**	6.76	0.00	6.76	8.08	0.00	8.08	5.93	0.00	5.93
	**2016**	6.84	0.00	6.84	9.04	0.00	9.04	5.51	0.00	5.51
	**2017**	6.97	0.00	6.97	9.98	0.00	9.98	5.16	0.00	5.16
	**2018**	7.97	0.00	7.97	10.93	0.00	10.93	6.44	0.00	6.44
	**2019**	8.17	0.00	8.17	10.97	0.00	10.97	6.66	0.00	6.66
	**2020**	9.41	0.00	9.41	12.64	0.00	12.64	7.60	0.00	7.60
	**2021**	11.14	0.00	11.14	14.78	0.00	14.78	8.58	0.00	8.58
Others	**2015**	4.70	0.00	4.70	9.28	0.00	9.28	1.80	0.00	1.80
	**2016**	5.06	0.00	5.06	9.36	0.00	9.36	2.41	0.00	2.41
	**2017**	4.59	0.00	4.59	9.38	0.00	9.38	1.73	0.00	1.73
	**2018**	4.44	0.00	4.44	8.50	0.00	8.50	2.04	0.00	2.04
	**2019**	3.96	0.00	3.96	7.88	0.00	7.88	1.67	0.00	1.67
	**2020**	4.78	0.00	4.78	9.41	0.00	9.41	2.07	0.00	2.07
	**2021**	4.80	0.00	4.80	8.99	0.00	8.99	2.14	0.00	2.14
	**2015**	6.33	0.00	6.33	7.13	0.00	7.13	5.57	0.00	5.57

**Diseases of pregnancy. childbirth. and perinatal period**	**2016**	5.80	0.00	5.80	6.41	0.00	6.41	5.23	0.00	5.23
	**2017**	4.93	0.00	4.93	5.29	0.00	5.29	4.57	0.00	4.57
	**2018**	5.12	0.00	5.12	5.69	0.00	5.69	4.53	0.00	4.53
	**2019**	5.32	0.00	5.32	5.94	0.00	5.94	4.66	0.00	4.66
	**2020**	5.59	0.00	5.59	6.16	0.00	6.16	5.02	0.00	5.02
	**2021**	6.40	0.00	6.40	6.18	0.00	6.18	6.58	0.00	6.58
Certain conditions originating in the perinatal period	**2015**	6.16	0.00	6.16	7.13	0.00	7.13	5.23	0.00	5.23
	**2016**	5.58	0.00	5.58	6.41	0.00	6.41	4.80	0.00	4.80
	**2017**	4.76	0.00	4.76	5.29	0.00	5.29	4.22	0.00	4.22
	**2018**	4.91	0.00	4.91	5.69	0.00	5.69	4.11	0.00	4.11
	**2019**	5.14	0.00	5.14	5.94	0.00	5.94	4.29	0.00	4.29
	**2020**	5.24	0.00	5.24	6.16	0.00	6.16	4.34	0.00	4.34
	**2021**	5.86	0.00	5.86	6.18	0.00	6.18	5.51	0.00	5.51
Others	**2015**	0.17	0.00	0.17	0.00	0.00	0.00	0.34	0.00	0.34
	**2016**	0.22	0.00	0.22	0.00	0.00	0.00	0.43	0.00	0.43
	**2017**	0.18	0.00	0.18	0.00	0.00	0.00	0.35	0.00	0.35
	**2018**	0.21	0.00	0.21	0.00	0.00	0.00	0.42	0.00	0.42
	**2019**	0.18	0.00	0.18	0.00	0.00	0.00	0.37	0.00	0.37
	**2020**	0.34	0.00	0.34	0.00	0.00	0.00	0.68	0.00	0.68
	**2021**	0.54	0.00	0.54	0.00	0.00	0.00	1.07	0.00	1.07
**Congenital malformations**	**2015**	1.84	0.03	1.82	1.97	0.04	1.93	1.74	0.01	1.73
	**2016**	1.82	0.05	1.77	1.91	0.06	1.85	1.76	0.04	1.72
	**2017**	1.73	0.05	1.68	1.87	0.04	1.82	1.60	0.07	1.53
	**2018**	1.68	0.05	1.64	1.97	0.05	1.93	1.42	0.04	1.37
	**2019**	1.46	0.05	1.41	1.48	0.06	1.42	1.45	0.05	1.40
	**2020**	1.47	0.07	1.40	1.58	0.08	1.51	1.38	0.06	1.31
	**2021**	1.51	0.07	1.44	1.71	0.07	1.65	1.31	0.07	1.24
Congenital malformations of the circulatory system	**2015**	1.82	0.00	1.82	1.93	0.00	1.93	1.73	0.00	1.73
	**2016**	1.77	0.00	1.77	1.85	0.00	1.85	1.72	0.00	1.72
	**2017**	1.68	0.00	1.68	1.82	0.00	1.82	1.53	0.00	1.53
	**2018**	1.64	0.00	1.64	1.93	0.00	1.93	1.37	0.00	1.37
	**2019**	1.41	0.00	1.41	1.42	0.00	1.42	1.40	0.00	1.40
	**2020**	1.40	0.00	1.40	1.51	0.00	1.51	1.31	0.00	1.31
	**2021**	1.44	0.00	1.44	1.65	0.00	1.65	1.24	0.00	1.24
Others	**2015**	0.03	0.03	0.00	0.04	0.04	0.00	0.01	0.01	0.00
	**2016**	0.05	0.05	0.00	0.06	0.06	0.00	0.04	0.04	0.00
	**2017**	0.05	0.05	0.00	0.04	0.04	0.00	0.07	0.07	0.00
	**2018**	0.05	0.05	0.00	0.05	0.05	0.00	0.04	0.04	0.00
	**2019**	0.05	0.05	0.00	0.06	0.06	0.00	0.05	0.05	0.00

	**2020**	0.07	0.07	0.00	0.08	0.08	0.00	0.06	0.06	0.00
	**2021**	0.07	0.07	0.00	0.07	0.07	0.00	0.07	0.07	0.00
**Adverse effects of medical and surgical care**	**2015**	0.18	0.00	0.18	0.25	0.00	0.25	0.12	0.00	0.12
	**2016**	0.12	0.00	0.12	0.17	0.00	0.17	0.08	0.00	0.08
	**2017**	0.14	0.00	0.14	0.17	0.00	0.17	0.12	0.00	0.12
	**2018**	0.13	0.00	0.13	0.19	0.00	0.19	0.10	0.00	0.10
	**2019**	0.09	0.00	0.09	0.13	0.00	0.13	0.06	0.00	0.06
	**2020**	0.11	0.00	0.11	0.13	0.00	0.13	0.10	0.00	0.10
	**2021**	0.10	0.00	0.10	0.09	0.00	0.09	0.11	0.00	0.11
Misadventures to patients during surgical and medical care	**2015**	0.05	0.00	0.05	0.10	0.00	0.10	0.01	0.00	0.01
	**2016**	0.06	0.00	0.06	0.07	0.00	0.07	0.06	0.00	0.06
	**2017**	0.06	0.00	0.06	0.10	0.00	0.10	0.04	0.00	0.04
	**2018**	0.05	0.00	0.05	0.09	0.00	0.09	0.03	0.00	0.03
	**2019**	0.07	0.00	0.07	0.10	0.00	0.10	0.05	0.00	0.05
	**2020**	0.03	0.00	0.03	0.05	0.00	0.05	0.02	0.00	0.02
	**2021**	0.03	0.00	0.03	0.01	0.00	0.01	0.03	0.00	0.03
Others	**2015**	0.13	0.00	0.13	0.15	0.00	0.15	0.11	0.00	0.11
	**2016**	0.06	0.00	0.06	0.10	0.00	0.10	0.03	0.00	0.03
	**2017**	0.08	0.00	0.08	0.07	0.00	0.07	0.08	0.00	0.08
	**2018**	0.08	0.00	0.08	0.10	0.00	0.10	0.07	0.00	0.07
	**2019**	0.02	0.00	0.02	0.04	0.00	0.04	0.01	0.00	0.01
	**2020**	0.08	0.00	0.08	0.09	0.00	0.09	0.08	0.00	0.08
	**2021**	0.07	0.00	0.07	0.07	0.00	0.07	0.07	0.00	0.07
**Injuries**	**2015**	74.57	74.57	0.00	128.13	128.13	0.00	29.13	29.13	0.00
	**2016**	69.46	69.46	0.00	119.34	119.34	0.00	27.15	27.15	0.00
	**2017**	64.37	64.37	0.00	110.27	110.27	0.00	25.45	25.45	0.00
	**2018**	62.27	62.27	0.00	106.02	106.02	0.00	24.77	24.77	0.00
	**2019**	61.61	61.61	0.00	105.37	105.37	0.00	24.27	24.27	0.00
	**2020**	53.87	53.87	0.00	93.67	93.67	0.00	19.84	19.84	0.00
	**2021**	56.74	56.74	0.00	96.27	96.27	0.00	22.10	22.10	0.00
Transport accidents	**2015**	15.04	15.04	0.00	23.77	23.77	0.00	7.40	7.40	0.00
	**2016**	15.57	15.57	0.00	24.61	24.61	0.00	7.75	7.75	0.00
	**2017**	13.32	13.32	0.00	20.67	20.67	0.00	7.02	7.02	0.00
	**2018**	13.42	13.42	0.00	20.79	20.79	0.00	6.87	6.87	0.00
	**2019**	15.02	15.02	0.00	23.55	23.55	0.00	7.48	7.48	0.00
	**2020**	11.77	11.77	0.00	18.98	18.98	0.00	5.38	5.38	0.00
	**2021**	12.56	12.56	0.00	19.64	19.64	0.00	6.17	6.17	0.00
Intentional self-harm	**2015**	16.49	16.49	0.00	29.31	29.31	0.00	5.39	5.39	0.00
	**2016**	14.58	14.58	0.00	26.11	26.11	0.00	4.62	4.62	0.00

	**2017**	14.91	14.91	0.00	26.34	26.34	0.00	5.05	5.05	0.00
	**2018**	13.94	13.94	0.00	25.25	25.25	0.00	4.10	4.10	0.00
	**2019**	13.23	13.23	0.00	24.33	24.33	0.00	3.66	3.66	0.00
	**2020**	11.87	11.87	0.00	21.53	21.53	0.00	3.59	3.59	0.00
	**2021**	11.57	11.57	0.00	20.53	20.53	0.00	3.72	3.72	0.00
Others	**2015**	43.04	43.04	0.00	75.05	75.05	0.00	16.34	16.34	0.00
	**2016**	39.31	39.31	0.00	68.62	68.62	0.00	14.78	14.78	0.00
	**2017**	36.14	36.14	0.00	63.26	63.26	0.00	13.38	13.38	0.00
	**2018**	34.90	34.90	0.00	59.99	59.99	0.00	13.80	13.80	0.00
	**2019**	33.36	33.36	0.00	57.50	57.50	0.00	13.14	13.14	0.00
	**2020**	30.23	30.23	0.00	53.16	53.16	0.00	10.87	10.87	0.00
	**2021**	32.61	32.61	0.00	56.10	56.10	0.00	12.20	12.20	0.00
**Alcohol-related and drug-related deaths**	**2015**	48.06	48.06	0.00	73.63	73.63	0.00	28.45	28.45	0.00
	**2016**	43.27	43.27	0.00	67.00	67.00	0.00	25.57	25.57	0.00
	**2017**	40.91	40.91	0.00	61.89	61.89	0.00	25.50	25.50	0.00
	**2018**	40.84	40.84	0.00	62.42	62.42	0.00	25.32	25.32	0.00
	**2019**	43.48	43.48	0.00	65.84	65.84	0.00	27.62	27.62	0.00
	**2020**	43.66	43.66	0.00	67.09	67.09	0.00	26.43	26.43	0.00
	**2021**	47.24	47.24	0.00	71.47	71.47	0.00	28.27	28.27	0.00
Alcohol specific disorders and poisonings	**2015**	46.21	46.21	0.00	70.80	70.80	0.00	27.53	27.53	0.00
	**2016**	42.16	42.16	0.00	65.27	65.27	0.00	25.04	25.04	0.00
	**2017**	39.89	39.89	0.00	60.36	60.36	0.00	24.95	24.95	0.00
	**2018**	39.86	39.86	0.00	60.88	60.88	0.00	24.83	24.83	0.00
	**2019**	42.56	42.56	0.00	64.47	64.47	0.00	27.10	27.10	0.00
	**2020**	43.02	43.02	0.00	66.14	66.14	0.00	26.05	26.05	0.00
	**2021**	46.56	46.56	0.00	70.52	70.52	0.00	27.84	27.84	0.00
Drug -related deaths	**2015**	1.85	1.85	0.00	2.84	2.84	0.00	0.92	0.92	0.00
	**2016**	1.11	1.11	0.00	1.74	1.74	0.00	0.53	0.53	0.00
	**2017**	1.02	1.02	0.00	1.53	1.53	0.00	0.55	0.55	0.00
	**2018**	0.98	0.98	0.00	1.54	1.54	0.00	0.49	0.49	0.00
	**2019**	0.92	0.92	0.00	1.38	1.38	0.00	0.52	0.52	0.00
	**2020**	0.65	0.65	0.00	0.95	0.95	0.00	0.38	0.38	0.00
	**2021**	0.68	0.68	0.00	0.95	0.95	0.00	0.43	0.43	0.00
**Provisional assignment of new diseases – COVID-19**	**2020**	18.08	18.08	0.00	25.11	25.11	0.00	14.12	14.12	0.00
	**2021**	83.91	83.91	0.00	95.76	95.76	0.00	76.07	76.07	0.00
